# From sudden stroke to heart transplant: unmasking restrictive cardiomyopathy in an adolescent: a case report

**DOI:** 10.1093/ehjcr/ytaf658

**Published:** 2025-12-18

**Authors:** José Luis López-Guillén, Mike Seed, Anne I Dipchand, Aamir Jeewa, Josh Griesman

**Affiliations:** Division of Cardiology, Labatt Family Heart Centre, The Hospital for Sick Children, University of Toronto, 175 Elizabeth St, Toronto, ON, Canada M5G 1E8; Division of Cardiology, Labatt Family Heart Centre, The Hospital for Sick Children, University of Toronto, 175 Elizabeth St, Toronto, ON, Canada M5G 1E8; Division of Cardiology, Labatt Family Heart Centre, The Hospital for Sick Children, University of Toronto, 175 Elizabeth St, Toronto, ON, Canada M5G 1E8; Division of Cardiology, Labatt Family Heart Centre, The Hospital for Sick Children, University of Toronto, 175 Elizabeth St, Toronto, ON, Canada M5G 1E8; Division of Cardiology, Labatt Family Heart Centre, The Hospital for Sick Children, University of Toronto, 175 Elizabeth St, Toronto, ON, Canada M5G 1E8

**Keywords:** Acute ischaemic stroke, Restrictive cardiomyopathy, Cardioembolic source, Paediatrics, Case report

## Abstract

**Background:**

Paediatric acute ischaemic stroke (AIS) is a challenging diagnostic entity. A comprehensive cardiac evaluation is essential in all paediatric AIS cases to rule out cardioembolic sources. We present the case of a teenage female with AIS who was found to have restrictive cardiomyopathy (RCM).

**Case summary:**

A 15-year-old female presented to the emergency department with left-sided paresis, hemi-neglect, and facial paralysis. Neuroimaging demonstrated a right middle cerebral artery infarct and mechanical thrombectomy was performed. Chest radiography revealed cardiomegaly and pulmonary oedema, and electrocardiogram (ECG) showed biatrial enlargement with repolarization abnormalities. Echocardiography revealed RCM, which was subsequently confirmed by cardiac magnetic resonance imaging, providing support for a cardioembolic source, and she received a heart transplant. Genetic testing ultimately identified a heterozygous pathogenic variant in *MYH7*.

**Discussion:**

Paediatric AIS is uncommon and frequently idiopathic, with cardioembolism accounting for ∼15% of adolescent cases. Restrictive cardiomyopathy, although rare, should be considered in the differential, as thromboembolic complications occur in up to one-third of patients, with nearly half of these involving the cerebral circulation. Importantly, stroke may be the first manifestation of the disease. Early cardiac screening, including ECG, chest radiography, N-terminal pro-B-type natriuretic peptide (NT-proBNP), and echocardiography, is crucial in the early diagnostic work-up of paediatric AIS. Prompt recognition of diastolic dysfunction and atrial enlargement may accelerate diagnosis and inform timely referral for heart transplantation. In short, paediatric AIS, though rare, requires thorough diagnostic evaluation to identify potential cardioembolic sources.

Learning points

- Restrictive cardiomyopathy (RCM) may first present as acute ischaemic stroke in children, emphasizing the need to consider cardiac causes in paediatric stroke evaluation.
- Severe atrial enlargement and diastolic dysfunction in RCM markedly increase the risk of thromboembolic complications, including cerebrovascular events.
- Early cardiac screening supports timely diagnosis of RCM, facilitates transplant referral, and improves outcomes in children with stroke of unclear origin.

## Introduction

Paediatric acute ischaemic stroke (AIS) is a rare condition, with an incidence of 2.5–13 per 100 000 children annually. The variability in presentation and low clinical suspicion often contribute to delayed diagnosis, increasing morbidity and mortality.^1^ The pathogenesis of AIS in the paediatric population differs from that in adults; 50% of paediatric cases occur in children without known vascular risk factors.^2^ Although cardioembolic events are relatively rare in adolescents,^3^ they should be considered in the differential diagnosis of ischaemic stroke, especially in cases without an apparent cause (see Supplementary material online, *Table S1*). Notably, AIS secondary to restrictive cardiomyopathy (RCM) remains underreported, with limited data available in the paediatric population.^4^

## Summary figure

**Table:** 

**12:00 p.m.**	Patient attempted to stand from a sitting position and noted sudden onset of weakness on the left side, rendering her unable to stand. Her mother immediately transported her to a local hospital for evaluation.
**1:08 p.m.**	Examination revealed left-sided hemiparesis and left-sided neglect, left facial droop, and hemisensory loss in addition to slurred speech.
**1:20 p.m.**	Non-contrast head CT showed features concerning for right MCA stroke in the M1/M2 territory (NIHSS 10, ASPECTS 10).
**1:42 p.m.**	Intravenous tissue plasminogen activator was administered. The patient remained haemodynamically stable. A transfer was initiated to our tertiary care centre for possible endovascular thrombolysis.
**4:17–5:30 p.m.**	Arrival at the Paediatric Intensive Care Unit.
	**Assessment pre-thrombectomy (NIHSS—12)**
	Item 1a—Level of Consciousness (LOC): 1 (/3 points)
	Item 1b—LOC Questions: 0 (/2 points)
	Item 1c—LOC Commands: 0 (/2 points)
	Item 2—Best Gaze: 0 (/2 points)
	Item 3—Visual: 1 (/3 points)
	Item 4—Facial Palsy: 2 (/3 points)
	Item 5a—Motor Left Arm: 2 (/4 points)
	Item 5b—Motor Right Arm: 0 (/4 points)
	Item 6a—Motor Left Leg: 0 (/4 points)
	Item 6b—Motor Right Leg: 0 (/4 points)
	Item 7—Limb Ataxia: 2 (/2 points)
	Item 8—Sensory: 1 (/2 points)
	Item 9—Best Language: 0 (/3 points)
	Item 10—Dysarthria: 1 (/2 points)
	Item 11—Extinction and Inattention (Neglect): 2 (/2 points)
**6:06–7:30 p.m.**	Mechanical thrombectomy performed. Three passes at M1 branch, achieving complete recanalization (residual non-flow limiting clot). Intra-procedural vasospasm and transient hypotension occurred.
**8:00 p.m.**	Post-thrombectomy neurological examination similar to baseline; persistent left-sided weakness, facial droop, and dysarthria noted.
**9:00 Pm**	Cardiac evaluation revealed abnormal ECG and chest X-ray (CXR). Echocardiogram revealed left atrial enlargement and diastolic dysfunction concerning for restrictive physiology with no presence of intra-cardiac clots.

## Case report

A previously healthy 15-year-old female presented to her local emergency department with sudden-onset left-sided weakness and facial numbness. Neurological examination revealed left-sided hemiparesis, left-hemisensory symptoms, left centrofacial palsy, and dysarthria. A head computed tomography (CT) scan and paediatric stroke scores, including the National Institutes of Health Stroke Scale (NIHSS) and Alberta Stroke Program Early CT Score (ASPECTS), were consistent with an AIS localized to the territory of the right middle cerebral artery (MCA). Alteplase was administered, and she was transferred to our tertiary care centre.

Upon arrival, she was afebrile, with a blood pressure of 98/44 mmHg, heart rate of 66 beats/min, respiratory rate of 19 breaths/min, and oxygen saturation of 94% in room air. Cardiac exam was largely unremarkable with no murmur detected; however, bilateral pulmonary crackles were noted on auscultation. Initial laboratory investigations showed normal complete blood count, electrolytes, arterial blood gas and renal function. Screening for thrombophilia and other prothrombotic conditions was also completed and found to be unremarkable.

**Figure 1:**
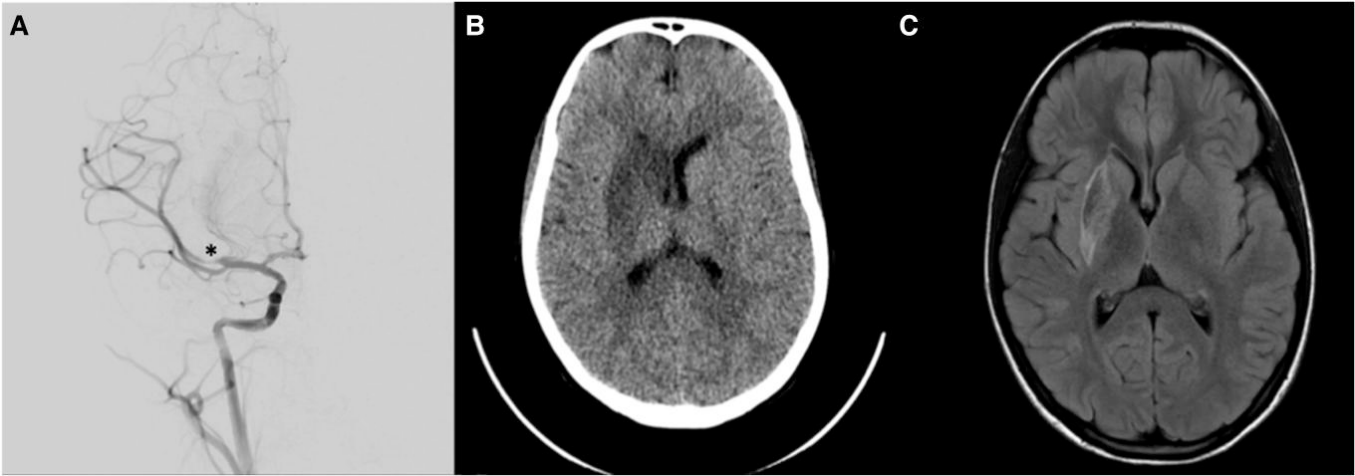
(*A*) Digital subtraction angiography, lateral projection, demonstrates abrupt occlusion of the M1 segment of the right middle cerebral artery (marked with *). (*B*) Non-contrast axial head computed tomography shows a region of hypoattenuation involving the right basal ganglia, as well as the anterior and posterior limbs of the internal capsule. Subtle loss of grey–white matter differentiation is seen in the affected regions, and there is early effacement of the ipsilateral anterior horn of the lateral ventricle. (*C*) Axial diffusion-weighted magnetic resonance imaging demonstrates restricted diffusion in the right basal ganglia and internal capsule, correlating with acute ischaemia. There is associated swelling and partial compression of the adjacent frontal horn of the right lateral ventricle, consistent with early mass effect.

**Figure 2:**
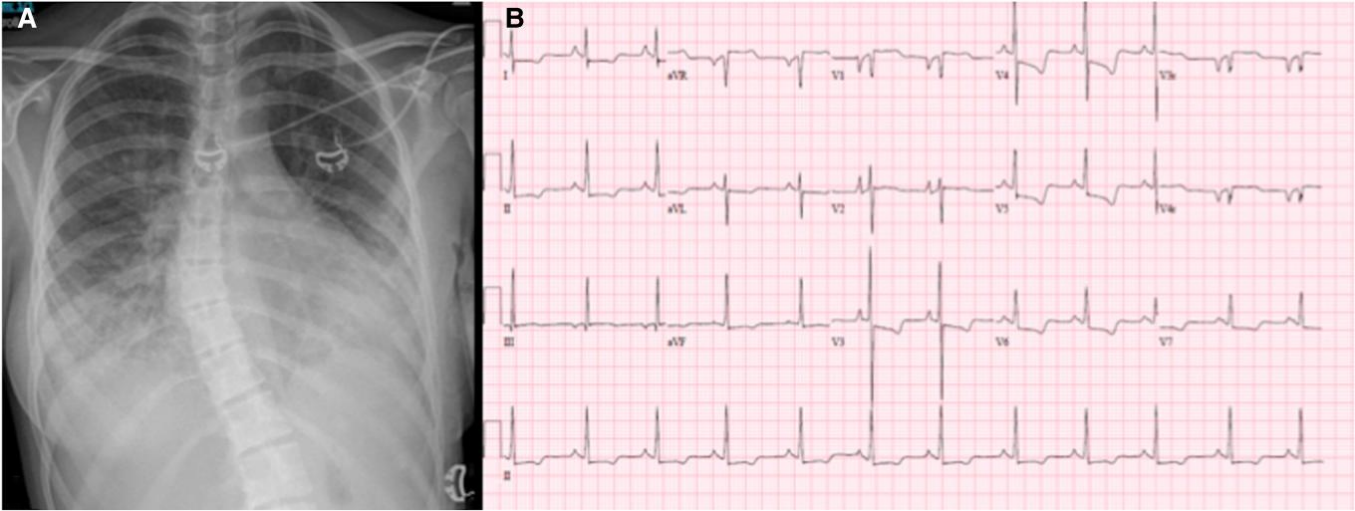
(*A*) Chest X-ray with moderate cardiomegaly, bilateral pleural effusions, and pulmonary oedema. (*B*). The 15-lead electrocardiogram demonstrates biatrial enlargement, with tall, peaked P waves in the inferior leads consistent with right atrial enlargement, and broad, bifid P waves in lead II with a negative terminal P-wave in V1 consistent with left atrial enlargement. There are also diffuse ST-T wave abnormalities (flattened to mildly inverted T waves across precordial and limb leads).

**Figure 3:**
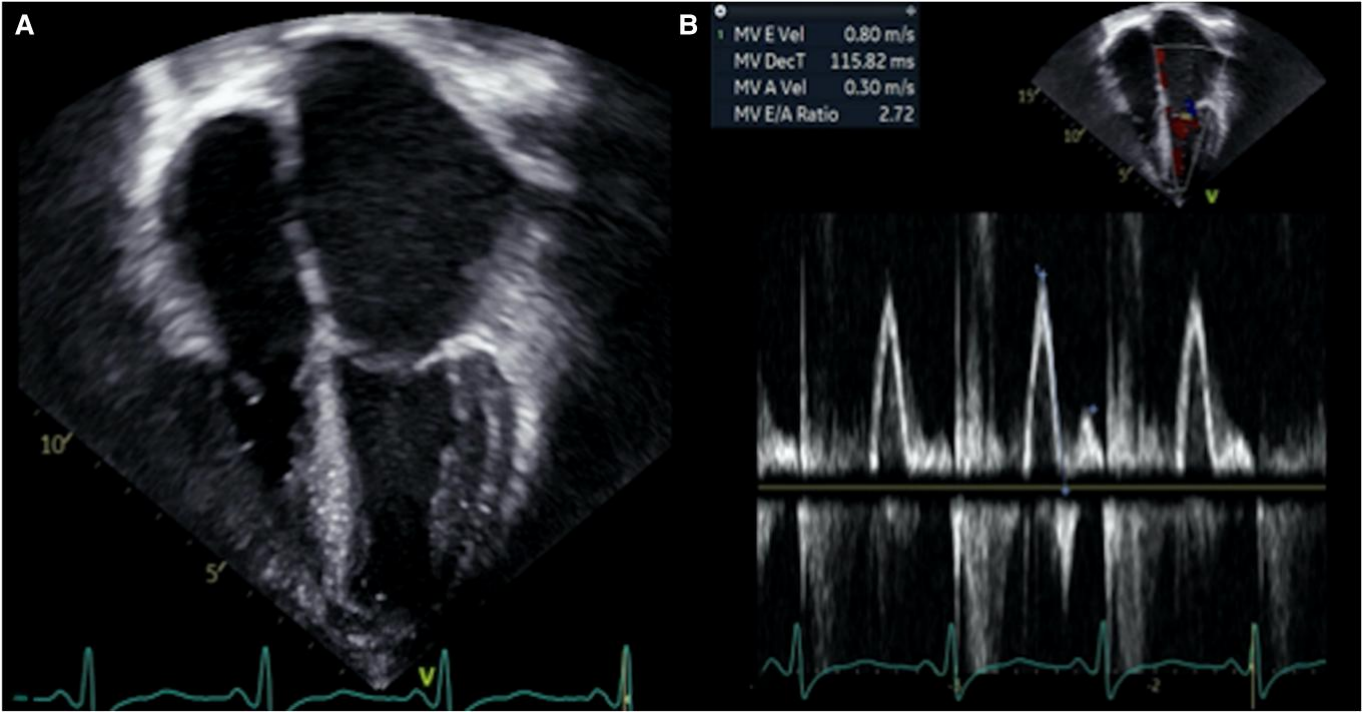
(*A*) Apical four-chamber echocardiographic view showing severe biatrial enlargement, with a markedly increased left atrial volume index of 68.7 mL/m^2^. The left ventricular chamber size remains within normal limits, highlighting the disproportionate degree of atrial dilatation relative to ventricular dimensions. (*B*) Pulsed-wave Doppler of mitral inflow showing an elevated *E*/*A* ratio (2.72) with a shortened deceleration time (116 ms) according to age values, findings consistent with chronically elevated left ventricular filling pressures and restrictive physiology.

**Figure 4:**
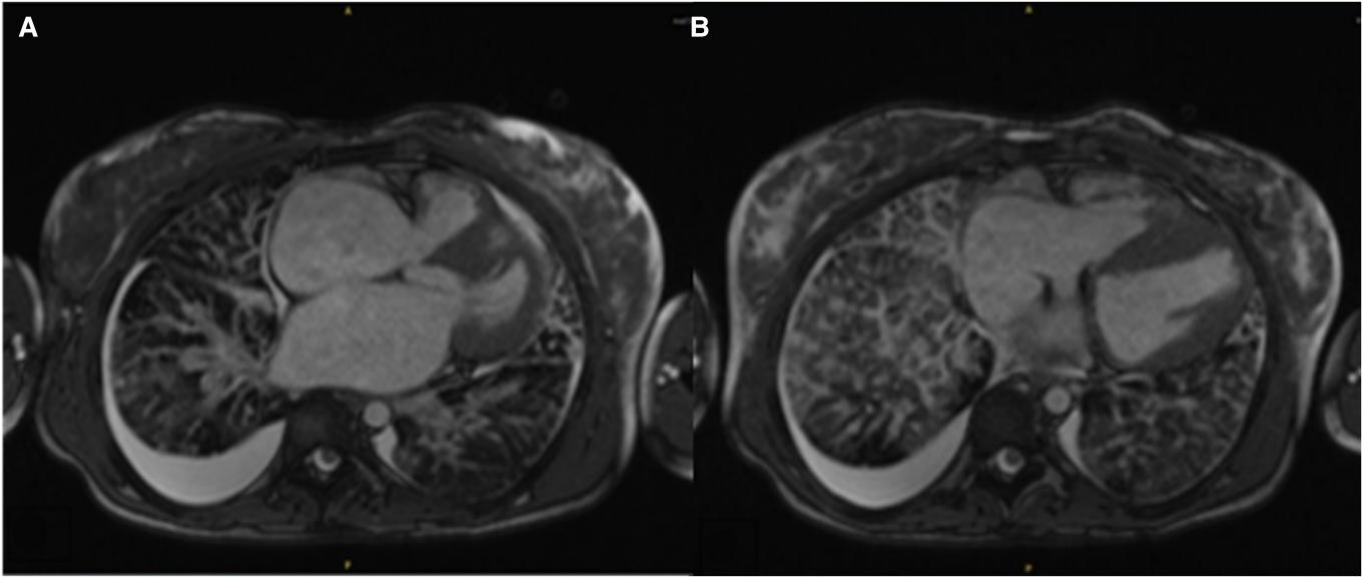
(*A*) Axial cardiac magnetic resonance imaging using steady-state free precession sequence demonstrating qualitatively severe biatrial dilation with reduced ventricular volumes (left ventricular end-diastolic volume index 65 mL/m^2^ and right ventricular end-diastolic volume index 71 mL/m^2^) and preserved biventricular function (left ventricular ejection fraction 63% and right ventricular ejection fraction 66%) in keeping with restrictive cardiomyopathy. (*B*) Associated findings include thickened interlobar fissures, interlobular septal thickening accompanied by areas of pulmonary lymphangiectasia and bilateral pleural effusions (right > left). These features reflect systemic venous and lymphatic congestion secondary to impaired diastolic filling.

Head CT and brain magnetic resonance imaging (MRI) revealed a right MCA territory infarct which was confirmed by angiography. She underwent urgent mechanical thrombectomy (*Figure 1*). Additional investigations included a CXR, which showed cardiomegaly and pulmonary oedema, and an electrocardiogram (ECG), which revealed biatrial enlargement and repolarization abnormalities (*Figure 2*). An echocardiogram completed after thrombectomy demonstrated severe left atrial enlargement and abnormal relaxation with preserved systolic function, consistent with RCM (*Figure 3*). Her N-terminal pro-B-type natriuretic peptide (NT-proBNP) levels were elevated at 15 000 ng/L, supporting a cardiac aetiology. Cardiac MRI findings further corroborated the diagnosis of RCM (*Figure 4*).

A heparin infusion was started for secondary prevention, given the severely dilated left atrium, suspected to be the source of the thrombus. A small non-obstructing thrombus in the MCA could not be retrieved and was noted on repeat imaging, which posed a risk for thrombotic recurrence, and she was therefore transitioned to warfarin. The patient demonstrated gradual and progressive recovery, with overall improvement in neurological deficits, except for persistent mild weakness in the left upper extremity. One month after presentation, she underwent a successful orthotopic heart transplant. Genetic testing ultimately revealed a pathogenic heterozygous mutation in *MYH7* gene (c.715G > A, p.Asp239Asn).

## Discussion

Approximately 15% of AIS cases in adolescents are cardioembolic in origin.^2^ Although rare, RCM represents a significant but often underrecognized cause of cardioembolic AIS in the paediatric population.^3^ Restrictive cardiomyopathy predisposes patients to thromboembolic events occurring in 10%–30% of patients; half of which are cerebrovascular events.^4^ Among the cardiomyopathies diagnosed in children, RCM is the least common but notable for its particularly poor prognosis, with a 2-year transplant-free survival rate of ∼50%.^5,6^ This condition is characterized by diastolic dysfunction, which leads to left atrial dilation and blood stasis, which creates a nidus for clot formation.^7^ While most thromboembolic events described in RCM occur after the diagnosis, they may occasionally be the first manifestation of the disease, as illustrated by our patient.

A high index of suspicion for a cardiac cause is essential in the evaluation of paediatric stroke.^1–3^ In this case, cardiac imaging was invaluable in diagnosing RCM, yet paediatric echocardiography may not always be readily available, especially in peripheral centres. Routine and easily accessible tests can therefore provide early clues to an underlying cardiac aetiology. In our patient, both ECG and CXR revealed abnormalities, and markedly elevated NT-proBNP levels further supported a cardiac source. These first-line investigations should be utilized early in the workup for paediatric stroke, and the ECG should be scrutinized for both functional and structural abnormalities, in addition to the presence or absence of arrhythmia. Importantly, while cardiac evaluation is essential, acute stroke management should never be delayed waiting for an echocardiogram. To the best of our knowledge, only one prior case of paediatric AIS preceding the diagnosis of RCM has been reported by Kulhari *et al*.^8^, who described a 9-year-old boy with a TNNI3 mutation (*Table 1*).

**Table 1:** Reported cases of ischaemic stroke preceding a diagnosis of restrictive cardiomyopathy in the paediatric population

	Patient age/sex	Stroke presentation	Imaging findings	Cardiac findings	Genetic testing	Treatment	Outcomes
**Current case**	15–year-old female	Left hemiparesis, left-hemisensory symptoms, left centrofacial palsy, and dysarthria	CT/MRI: right MCA infarct	ECG: biatrial enlargement and repolarization abnormalities, CXR: bilateral pulmonary oedema NT-proBNP 15 000 ng/L, Echo: severe left atrial enlargement, restrictive filling pattern CMR: confirmed RCM	MYH7 heterozygous pathogenic variant	Alteplase, mechanical thrombectomy, anticoagulation (warfarin), heart transplantation	Gradual neurological recovery, successful orthotopic transplant, no thromboembolic sequelae
**Kulhari *et al*.^8^**	9-year-old boy	Left hemiparesis, hemihypesthesia, and dysarthria	CT/MRI: right MCA infarct	ECG: biatrial enlargement Echo: severe bilateral atrial enlargement with normal ventricular size and function, CMR: confirmed RCM	Missense mutation in the TNNI3 gene	Mechanical thrombectomy, anticoagulation (dabigatran), heart transplantation	Neurologically stable, successful orthotopic transplant, no thromboembolic sequalae

Clinical, diagnostic, genetic, and therapeutic features.

CT, computed tomography; CMR, cardiac MRI; MRI, magnetic resonance imaging; MCA, middle cerebral artery; RCM, restrictive cardiomyopathy.

Restrictive cardiomyopathy in children is predominantly defined by diastolic dysfunction, where impaired ventricular relaxation and increased myocardial stiffness lead to elevated filling pressures despite preserved systolic function.^5^ Echocardiographic markers such as an increased *E*/*A* ratio, shortened deceleration time, reduced tissue Doppler *e*′ velocities, and elevated *E*/*e*′ ratio can provide important diagnostic clues.^6^ However, in the paediatric population, these parameters are highly age-dependent and technically challenging to interpret, as normal ranges vary with growth and maturation, and reliable acquisition requires specialized expertise. For this reason, a combination of findings, particularly when severe biatrial enlargement is present, adds greater diagnostic weight. Diastolic dysfunction not only explains clinical features such as respiratory symptoms, fatigue, or hepatomegaly, but also confers a high risk of thromboembolic events, including stroke.^7^ Early identification of diastolic dysfunction in paediatric AIS therefore supports timely recognition of RCM and facilitates referral for advanced management, including anticoagulation, genetic evaluation, and consideration for transplantation.

Genetic testing also plays a crucial role in the evaluation of RCM, providing definitive diagnostic confirmation and valuable prognostic information.^9^ Identifying pathogenic variants, such as those in the *MYH7* gene, can guide personalized management strategies, risk prognostication, and family screening.^10^ Both our case and the report by Kulhari *et al.*^8^ emphasize the critical role of genetic mutations in the pathogenesis of RCM associated with paediatric AIS. Furthermore, the genetic heterogeneity of RCM underscores the importance of comprehensive molecular testing in children presenting with stroke and cardiomyopathy.^6,9^ Together, these case reports underscore how genetic insights, combined with clinical and imaging findings, can guide multidisciplinary management to optimize cardiac outcomes in this population.

To conclude, we highlight the importance of maintaining a broad differential diagnosis in paediatric AIS and reinforce the need for cardiac screening in children presenting with stroke. In patients with RCM, thromboembolic events are not uncommon and may rarely present as the initial manifestation of the disease.

## Lead author biography


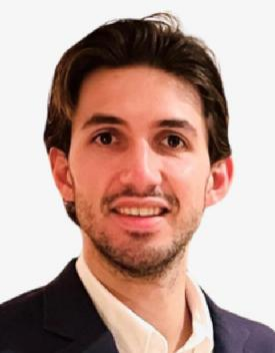


As a Paediatric Cardiologist, his scholarly and clinical interests focus on the investigation of cardiomyopathies, channelopathies, and the development and optimization of advanced heart failure therapeutics. His long-term academic objective is to integrate subspecialty clinical expertise with a systematic, mechanistic examination of the bidirectional interactions between the cardiovascular and neurological systems. By elucidating these complex pathophysiological relationships, he seeks to inform innovative diagnostic strategies and therapeutic interventions, thereby improving outcomes in paediatric populations affected by these life-threatening disorders.

## Supplementary Material

ytaf658_Supplementary_Data

## Data Availability

All data generated or analysed during this study are included in this published article (and its online Supplementary material).

## References

[1] Gao L, Lim M, Nguyen D, Bowe S, MacKay MT, Stojanovski B, et al The incidence of pediatric ischemic stroke: a systematic review and meta-analysis. Int J Stroke 2023;18:765–772. 36691675 10.1177/17474930231155336

[2] Mayne EW, Mailo JA, Pabst L, Pulcine E, Harrar DB, Waak M, et al Pediatric stroke and cardiac disease: challenges in recognition and management. Semin Pediatr Neurol 2022;43:100992. 36344023 10.1016/j.spen.2022.100992 PMC9719802

[3] Yu MY, Caprio FZ, Bernstein RA. Cardioembolic stroke. Neurol Clin 2024;42:651–661. 38937034 10.1016/j.ncl.2024.03.002

[4] Price JF, Jeewa A, Denfield SW. Clinical characteristics and treatment of cardiomyopathies in children. Curr Cardiol Rev 2016;12:85–98. 26926296 10.2174/1573403X12666160301115543 PMC4861947

[5] Lorenzo M, Lynch A, Ashkanase J, Fazari L, George K, Arathoon K, et al Symptomatic presentation influences outcomes in pediatric restrictive cardiomyopathy. Front Pediatr 2023;11:1264751. 37928350 10.3389/fped.2023.1264751 PMC10620919

[6] Arbelo E, Protonotarios A, Gimeno JR, Arbustini E, Barriales-Villa R, Basso C, et al 2023 ESC guidelines for the management of cardiomyopathies. G Ital Cardiol (Rome) 2023;24:e1–e127. 10.1714/4127.41209 37901944

[7] Ishida H, Narita J, Ishii R, Suginobe H, Tsuru H, Wang R, et al Clinical outcomes and genetic analyses of restrictive cardiomyopathy in children. Circ Genom Precis Med 2023;16:382–389. 37377035 10.1161/CIRCGEN.122.004054

[8] Kulhari A, Dorn E, Pace J, Alambyan V, Chen S, Wu OC, et al Acute ischemic pediatric stroke management: an extended window for mechanical thrombectomy? Front Neurol 2017;8:634. 29238322 10.3389/fneur.2017.00634 PMC5712569

[9] Musunuru K, Hershberger RE, Day SM, Klinedinst NJ, Landstrom AP, Parikh VN, et al Genetic testing for inherited cardiovascular diseases: a scientific statement from the American Heart Association. Circ Genom Precis Med 2020;13:e000067. 32698598 10.1161/HCG.0000000000000067

[10] Zhao Y, Liu S, Mo H, Hua X, Chen X, Zhang Y, et al MYH7 mutations in restrictive cardiomyopathy. JACC Adv 2025;4:101693. 40286359 10.1016/j.jacadv.2025.101693 PMC12102527

