# Supplementary material for: From sudden stroke to heart transplant: unmasking restrictive cardiomyopathy in an adolescent: a case report
Source: Eur Heart J Case Rep. 2025 Dec 18;10(1):ytaf658. doi: 10.1093/ehjcr/ytaf658 (PMC12798805; doi:10.1093/ehjcr/ytaf658)
Supplement: ytaf658_Supplementary_Data [file ytaf658_supplementary_data.docx]

| **Cardioembolic Source** | **Description** | **Examples / Associated Conditions** |
| --- | --- | --- |
| **Cardiomyopathies** | Structural or functional myocardial disorders leading to thrombus formation. | **Restrictive cardiomyopathy (RCM),** dilated cardiomyopathy (DCM), hypertrophic cardiomyopathy (HCM). |
| **Congenital heart disease (CHD)** | Abnormal cardiac anatomy increasing embolic risk, especially with shunts. | Atrial septal defect (ASD), patent foramen ovale (PFO), cyanotic CHD. |
| **Arrhythmias** | Irregular heart rhythms causing stasis and atrial thrombus. | Atrial flutter/fibrillation, supraventricular tachycardia (SVT), ventricular tachycardia (VT). |
| **Intracardiac thrombi or masses** | Clots or tumors within cardiac chambers that can embolize. | Left atrial/ventricular thrombus, atrial myxoma. |
| **Valvular heart disease** | Structural valve abnormalities or infections promoting thromboembolism. | Rheumatic heart disease, infective endocarditis. |
| **Myocarditis or pericarditis** | Inflammatory processes with potential thromboembolic complications. | Viral myocarditis, autoimmune pericarditis. |
| **Genetic syndromes** | Inherited conditions that predispose to cardiac abnormalities and embolism. | Marfan syndrome, Loeys-Dietz syndrome, MYH7-related cardiomyopathy. |
| **Cardiac devices** | Foreign bodies increasing thrombogenicity. | Mechanical and prosthetic valves, pacemaker leads, ventricular assist devices (VADs). |
| **Post-operative state** | Increased embolic risk following cardiac surgery or catheter-based interventions. | Fontan procedure, septal defect repair. |

**Supplementary Table 1:** Common cardioembolic sources of acute ischaemic stroke in paediatric patients.
